# Experimental noise and light pollution alter prey detection in a nocturnal bird of prey

**DOI:** 10.1111/1365-2656.70062

**Published:** 2025-05-21

**Authors:** Arianna Passarotto, Chiara Morosinotto, Patrik Karell

**Affiliations:** ^1^ Evolutionary Ecology Unit, Department of Biology Lund University Lund Sweden; ^2^ Universidad de Sevilla Sevilla Spain; ^3^ Bioeconomy Research Team, Novia University of Applied Sciences Raseborg Finland; ^4^ Department of Biology University of Padova Padova Italy; ^5^ National Biodiversity Future Center (NBFC) Palermo Italy; ^6^ Present address: School of Biodiversity, One Health and Veterinary Medicine, University of Glasgow University avenue, Graham Kerr Building Glasgow G128QQ UK

**Keywords:** acoustic predators, artificial light at night, environmental change, hunting behaviour, interactive effects, traffic noise, urbanisation

## Abstract

1. Urban expansion has led to ever increasing noise and light pollution, which impairs the audio‐visual perception of wild animals and drives changes in key activities and behaviours.
2. Nocturnal predators may be especially affected in detecting prey, with potentially dramatic consequences for their fitness. However, the combined effects of noise and light pollution on predator performance are still mostly unstudied.
3. We experimentally exposed tawny owls (*Strix aluco*), nocturnal acoustic raptors, to traffic noise and artificial light. We provided both visual and acoustic prey cues to assess potential non‐additive effects among multiple sensory stressors on the ability to detect prey.
4. We found that, in control conditions, owls responded equally to both acoustic and visual prey cues. In contrast, noise and light individually decreased owls' ability to locate acoustic, but not visual, prey cues. When owls were exposed to noise and light combined also visual detection worsened, but not beyond the additive expectation. Conversely, the presence of light seemingly buffered the negative impact of noise on acoustic detection, suggesting an antagonistic interaction.
5. Our findings show that both anthropogenic noise and light affect the hunting behaviour of a nocturnal avian predator, but with a stronger effect on acoustic than visual detection, suggesting that the magnitude of their disruptive impact might depend on the type of prey cue. This implies that sensory pollution might lead to increased reliance on sight‐oriented hunting strategies. Importantly, our study shows that the co‐occurrence of noise and light can have complex and unexpected impacts on behaviour, underscoring the importance of examining sensory pollution in a multimodal context.

## Graphical abstract

The study explores combined effects of noise and light pollution in a nocturnal avian predator and shows that both noise and light affect owls' ability to detect prey acoustically but not visually. Lower prey detection might decrease hunting success and fitness, and eventually lead to a shift in hunting strategies.

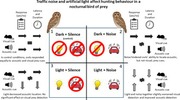

## 1 INTRODUCTION

Urbanisation is increasing at an unprecedented pace and is considered one of the main threats contributing to the current biodiversity crisis (Johnson et al., 2017). It is accompanied by a new set of stressors that pose novel challenges to wildlife, including, but not limited to, light and noise associated with human activities. Both stressors have been the target of extensive work by conservation and evolutionary biologists because they are considered sensory pollutants (Halfwerk & Slabbekoorn, 2015), as they alter the natural audio‐visual landscape and modify animals' sensory perception, with multifaceted impacts on organisms and, eventually, on populations (Dominoni, Halfwerk, et al., 2020; Gaston et al., 2013; Swaddle et al., 2015). Despite growing research effort, the magnitude of animal behavioural and physiological responses to light and noise pollution is still to be unravelled (Senzaki, Barber, et al., 2020; Tervo et al., 2023). In particular, the separate and combined effects of sensory pollutants on wildlife perception remain largely unexplored (Senzaki, Barber, et al., 2020).

The pervasive effects of both artificial light at night (hereafter ALAN) and anthropogenic noise (hereafter ‘noise’) affect a multitude of different aspects of animals' life in many taxa (e.g. Longcore & Rich, 2004; Slabbekoorn et al., 2018). Noise disrupts acoustic stimuli, also extending to remote areas far from the source of the disturbance (Buxton et al., 2017; Tervo et al., 2023), and is associated with an increased perception of risk. This often leads to the abandonment of usual sites (reviewed in Slabbekoorn et al., 2018), which is reflected in changes in distribution and community composition (Senzaki, Kadoya, et al., 2020). Even when animals do not leave, they can experience reduced fitness because of physiological stress (Kleist et al., 2018) and disruption in behaviour and performance due to masking or distraction. For instance, this can reduce foraging efficiency (Mason et al., 2016; Siemers & Schaub, 2011) and impair intraspecific communication (Butler & Maruska, 2020; Grade & Sieving, 2016).

Similarly, ALAN disrupts visual channels, impairing visually oriented behaviours of nocturnal organisms (Elgert et al., 2020) and obscuring important cues used for orientation at night (Burt et al., 2023; Foster et al., 2021). In addition, the spectra emitted by artificial lights, especially by LEDs, overlap natural light wavelengths (Davies & Smyth, 2018), which regulate circadian and seasonal activity rhythms through complex physiological processes (Dominoni, 2015). Therefore, constant exposure to ALAN can lead to stratified physiological, morphological and even genetic changes, cascading into behavioural and fitness impacts (Dominoni, 2015). In diurnal animals, light pollution interferes with sleep by prolonging the activity period and shifting time allocation for key activities (e.g. breeding behaviour, Dominoni et al., 2013), with high energetic costs due to restlessness (Raap et al., 2015). By disrupting biological rhythms, ALAN removes temporal partitioning between species with different activity rhythms, thereby creating novel interspecific competition, with significant repercussions on populations and ecosystems (Davies & Smyth, 2018; Longcore & Rich, 2004).

Although noise and light pollution largely co‐occur and trigger similar responses such as modulation of space use, activity rhythm and behaviour, they are mostly investigated separately (Dominoni, Smit, et al., 2020; Raap et al., 2017; Senzaki, Barber, et al., 2020). Consequently, we lack knowledge of their combined effects on species behaviour (Dominoni, Smit, et al., 2020; McMahon et al., 2017; Smit et al., 2022). In particular, it remains unclear whether noise and ALAN in concert evoke a stronger (synergistic) or a weaker (antagonistic) response in nocturnal species, which would indicate that their effects may not simply add up, as expected under an additive model, but that light and noise interact and influence each other's effects (Hale et al., 2017; Wilson et al., 2021). Noise is known to hinder those nocturnal taxa that rely on acoustic cues to move and forage at night, like bats and owls, describing patterns comparable to those observed in diurnal species (Senzaki et al., 2016; Siemers & Schaub, 2011). In contrast, the effects of ALAN on nocturnal wildlife are less understood (Dominoni, 2015; Elgert et al., 2020; Le Tallec et al., 2013) and appear highly species‐specific (Voigt et al., 2021), with maladaptive responses (Elgert et al., 2020) alongside positive, attractive effects (Rodríguez et al., 2021). These differing single patterns suggest that some species cope better than others with one or both stressors (Gomes et al., 2016; Voigt et al., 2021), indicating that combined effects might deviate more often than previously thought from additive expectations.

In this context, nocturnal predators are of particular relevance because they occupy the highest levels of the trophic web, playing a crucial role in ecosystem functioning. Their hunting success relies on the detection of subtle biological cues, making them especially vulnerable to sensory pollution (Konishi, 1973; Taylor, 2009). Moreover, nocturnal predators are often territorial and sedentary, which may promote the occurrence of coping strategies that improve hunting efficiency under the permanent changes brought by anthropogenic stressors (Gomes et al., 2016). Similarly to diurnal bird species that adjust song frequencies (Nemeth & Brumm, 2010) or singing timing (Gil et al., 2015) to overcome noise interference on communication, nocturnal predators might deal with sensory pollutants by either shifting their attention to other cues or by using different sensory channels, as observed in bats (Gomes et al., 2016; Voigt et al., 2021). Formal tests of the combined effects of noise and ALAN on hunting performance in nocturnal predators are needed to assess the generality of animal responses and understand how sensory pollutants interact and induce changes in hunting strategies and, ultimately, alter predator–prey dynamics (Komine et al., 2020; Villalobos‐Jiménez et al., 2017).

Here, we used a full‐factorial experimental approach to test for separate and combined effects of ALAN and noise on prey detection using captive‐reared tawny owls (*Strix aluco*). The tawny owl is a widespread forest‐dwelling nocturnal bird of prey with high site tenacity (Passarotto et al., 2023) and a distribution range which often intersects urbanised environments (Ratajc et al., 2023). Typically, tawny owls are ambush predators that locate prey via relevant acoustic cues (Martin, 1986), mainly preying on small mammals (Karell et al., 2021). In urban areas and in poor small mammal conditions, tawny owls can be opportunistic and include in their diet high percentages of birds, frogs and insects (Karell et al., 2021; Zalewski, 1994), which might be likely captured using variable (visual) cues, although this aspect of tawny owl hunting behaviour has not been explored much (Martin, 1986). These features make tawny owls particularly suitable to investigate whether and how sensory pollutants may modify owls' preference for certain types of cues and, consequently, their hunting habits. Previous empirical studies on other owl species have shown that noise impairs their hunting efficiency (Mason et al., 2016; Senzaki et al., 2016), but no study has assessed the single and combined effects of ALAN on owl hunting strategy so far.

The complexity of the possible interactive effects makes it difficult to cast univocal predictions (Dominoni, Halfwerk, et al., 2020; Dominoni, Smit, et al., 2020; Hale et al., 2017) and here we aim to test two main scenarios. On the one hand, ALAN and noise are expected to be equally harmful for a nocturnal predator as both may be perceived as disturbing because they prevent efficient information gathering and may promote adverse stress‐mediated responses and modify owl behaviour (Orlando & Chamberlain, 2023). Under this scenario, we hypothesise that ALAN and noise together would amplify each other's effects and induce a stronger negative response in owl prey detection. On the other hand, in a species with an acoustically driven hunting strategy, noise might disrupt behaviour more strongly than ALAN owing to the hindering effects on hearing and attention (Senzaki et al., 2016). Accordingly, we anticipate an overriding effect of noise on light. Additionally, the increase in environmental light might even be perceived as beneficial and constitute a resource, for example by increasing visibility during hunting (Gaston et al., 2013; Rodríguez et al., 2021). To formally test for a different use of perceptual cues in the presence of both stressors, we provided owls with both acoustic and visual prey cues. We predict that owls would mostly rely on visual cues in noise‐ and light‐polluted environments at the expense of acoustic cues to compensate for the masking and distracting effects of noise and to take advantage of artificial light.

## 2 MATERIALS AND METHODS

### 2.1 Study system and experimental facilities

The study was performed between February and March 2021 and 2022 with 38 captive‐reared tawny owls (19 in 2021 and 19 in 2022) held in purpose‐built outdoor aviaries at Stensoffa Biological Station of the Department of Biology, Lund University, in Southern Sweden. Stensoffa field station is situated in a quiet area in the countryside, ca. 300 m south of a minor local road and ca. 4 km north of the closest highway. At the station there is no artificial lighting during the night, except for a security light at the entrance gate that activates through a motion sensor, and the closest village is located ca. 3 km southwest (Figure S1).

Individuals used in the experiments were collected in the field from a long‐term monitored nest box population shortly prior to the fledging period, and kept in the aviaries until the subadult phase, when owls were successfully released into empty territories in the origin population in Skaraborg, Sweden (58.2528° N, 13.0596° E). Experiments were performed with subadult owls, which are fully independent and whose hunting skills are already developed (Sunde, 2008). During captivity, human contact was reduced to the time needed to feed, measure and sample the owls, and the perimeter of the structure was shielded with fabric to prevent habituation (Figure S2A).

At the biological station, each owl was housed in an individual aviary sized 3 × 6 × 3 m (width, length, height) and provided with a fully sheltered area and an equally sized open area, as well as several perches distributed between the two different parts. The experimental aviary was located at the end of the row oriented towards the countryside, on the opposite side of the entrance to the facility, but still with two adjacent aviaries (Figure S2A). To avoid any visual contact between the owls during the experiments, we covered both sides of the experimental aviary with black fabric.

The ethical permits for this work have been approved by the Swedish Board for animal experiments (permit number 5.8.18–06007/2019). Everyone working with the owls has passed the required ethical course in laboratory animal science and Swedish legislation and ethics at Lund University.

### 2.2 Experimental set‐up

#### 2.2.1 Light–Noise pollution treatment

The overarching goal of this study was to experimentally determine single and combined effects of anthropogenic noise and light on hunting behaviour in a nocturnal bird of prey. To this end, we applied a full‐factorial design with four treatments: (i) control with no manipulation (darkness and silence, DS), (ii) darkness and traffic noise (DN), (iii) artificial light and silence (LS) and (iv) light and noise (LN). The treatments were performed in a slightly different order in the 2 years: DS and LN were always the first and the last treatment respectively, while LS and DN were performed as second treatments, respectively, in 2021 and in 2022. We tested several owls per night, one at a time, grouping them in two randomised nights/groups for each trial, one group/night of 10 owls and another group/night of 9 owls, for a total of 16 nights (8 nights for each year). All 38 owls received the four treatments and were therefore used as their own control. The trials were scheduled to be conducted in consecutive nights, or as closely after each other, depending on optimal weather conditions (i.e. clear nights with no wind or only gentle breeze, and no precipitation). Trials started after sunset, when tawny owls typically start their activity (Southern, 1970).

To record videos of owls' behaviour during the experiment, we used two infrared cameras (BOLYGUARD Model: BG584 Series): one was placed above the entrance door of the experimental aviary to record the behaviour of owls perching in the open area, while a second camera was hung on the opposite side to record the behaviour of owls when perching in the sheltered area (Figure S2B).

The noise treatment consisted of a freely downloadable 1‐min mp3 file of actual road traffic noise with multiple car, motorcycle and truck sounds on a highway at night (https://www.partnersinrhyme.com/soundfx/carsoundfx.shtml). The recording did not contain horns, ambulance sirens or other sudden high‐pitched sounds. To ensure an equal distribution of the sound, the speaker playing the recording (ROXCORE BEAT 5.0) was placed on a support (1.5 m high pole) at 12 m outside in front of the experimental aviary (Figure S2B). The volume was set at the maximum level, ensuring inside the aviary variable levels of noise ranging from 60 to 45 dbA (according to the different sound levels in the recording), which are described as normal increase and decrease in noise levels during the night around urbanised areas (Smit et al., 2022). During the treatments with noise (i.e. DN and LN), the recording was played uninterruptedly for the entire duration of the trials, and owls were thus tested at random moments of the recording.

The light treatment consisted of a LED lamp (natural white, BILTEMA Art. 46‐3136) commonly used to illuminate small porches or private garden paths, attached to the roof of the experimental aviary between the open and the covered area at a height of about 3 m and about 1.5 m from the perch (Figure S2B). The lamp had a lighting angle of 110° and 1600 lumens brightness and was oriented downward towards the ground without directly illuminating the area where the prey cues were placed (see below). Light intensity varied from ca. 80 lux where the light beamed to ca. 40 lux in the area of the cues and reproduced elevated light conditions tawny owls likely experience in urban environments in proximity to lighting spots (Hale et al., 2015; Reusch et al., 2024).

To assess whether individuals behaved consistently (Bell et al., 2009), we repeated the treatments twice on a randomly chosen subset of tawny owls. In 2021, we repeated all four treatments on 10 owls, while in 2022 we repeated the treatments as follows: the treatments DS and LS were repeated on 10 owls, whereas the treatments DN and LN were repeated on 9 different owls, summing 29 owls (76.3%) tested multiple times. Repetitions were carried out on consecutive nights after completing the main trials and followed the same treatment order used for the main trials (see above). The order in which the owls were tested within each trial was randomised.

#### 2.2.2 Visual versus acoustic prey cue

Another aim of our study was to assess whether light pollution may elicit a higher response to visual cues at expense of acoustic cues in an acoustically oriented nocturnal predator. Therefore, during every trials, we provided the owls with two different stimuli: a visual prey cue and an acoustic prey cue. Overall, we exposed each owl to four acoustic cues and four visual cues, for a total of eight alternated cues/stimuli of 30 s each. The cues were shown/played after 2 min from the release in the experimental aviary (i.e. acclimation time to reduce the stress of the capture) always in the same order, and at intervals of 20 s from each other. Thus, each trial lasted 9 min (2 min of settling +7 min of stimulation).

As an acoustic prey cue, we used a freely downloadable recording (https://www.fesliyanstudios.com/sound‐effects‐search.php?q=Rat‐Mouse‐Running‐A2) with a set of rustling sounds mimicking those naturally produced by mammal prey when moving in the undergrowth. The recording, an 18‐s mp3 file, was played in a loop during the 30 s of acoustic stimulation, using different starting points to minimise pseudoreplication issues. We placed two portable Bluetooth speakers on the ground in the open area, facing each other to alternate left and right side (Figure S2B), hiding them under neutral‐coloured material to avoid bias in owls' responses. The speakers (ROXCORE BEAT 5.0 on the left and BILTEMA 5.0 on the right) had similar technical characteristics and were connected to two different smartphones to ensure independent remote functioning. Regardless of the treatment, the recording was played at ca. 30–35 dB SPL, which is the range of frequencies used in another study testing the effect of traffic noise on owls' foraging efficiency (Senzaki et al., 2016).

As a visual prey cue, we used a dead grey furred laboratory mouse of about 18 grams, identical to those used to feed the owls. The body mass largely overlaps the preferred prey size reported for the species (Karell et al., 2021). During the captive period, owls were mostly fed one‐day‐old dead rooster chicken, but mice were provided regularly every 7–10 days to diversify their diet. The mouse was placed in front of the outer perch and tied at both ends of the body to a fishing line, whose ends were in turn tied to two handles so that the observer pulled one of the ends of the string, alternating movement from left to right, to imitate the natural movement of a mouse. The mouse was dragged inside a box after each movement to hide it and was substituted regularly in case of wear. The owls were not fed the day before a trial, and between the trials, the owl only received 1/3 of the normal food supplement to encourage their response.

### 2.3 Behavioural variables

After concluding the experiment, we extracted from the videos three focal behaviours: responsiveness, latency and duration of the response. To do this, each prey cue, both acoustic and visual, was considered as a single event, producing eight observations for each owl during every treatment. In total, we recorded 1216 observations (608 in 2021 and 608 in 2022). We extracted the same focal behavioural variables for the repetitions, tallying 624 observations (320 in 2021 and 304 in 2022).

We defined responsiveness as a binary variable (yes/no), describing whether an owl reacts to a prey cue or not. We considered an owl reacted to a cue only when the owl's gaze was unambiguously oriented towards one of the dummy prey: for acoustic cues, we considered only those cases where owls successfully located the source of the rustling sounds (i.e. the speakers), whereas for visual cues we assigned ‘yes’ when the owl clearly looked at the mouse moving. Responses can be considered as an actual detection and not as the owls were just ‘listening’ to the sound without really looking at its source because of the angle of the head when oriented towards the cues, given by the position of both the speakers and the mouse in relation to the perch. Owls' response was nearly always identifiable; only in three cases we assigned ‘NA’ because the reaction was unclear, summing 1213 observations (99.8%). In repeated groups, we were not able to unambiguously identify the response in two cases, thus summing 622 observations (99.7%).

We defined latency as the time until an owl's first reaction to a cue, either visual or acoustic (Bateson & Martin, 2007). Accordingly, we extracted an individual latency value for both visual and acoustic cues for the four treatments. If an owl did not react to a specific cue at all during one of the trials, no latency value was assigned. Similarly, the duration of the response was defined as the total time an owl looked at a cue during the trials and was recorded as a proxy of the interest of an individual in a cue (Bateson & Martin, 2007). Both the latency and the duration were recorded in seconds using a stopwatch and, to minimise observer error, they were taken twice and then averaged. In the case an owl looked at the same cue (i.e. during the 30 s of exposure to a cue) several times, we considered a ‘cumulative’ duration, which is the sum of the durations of all those times. Multiple reactions at the same cue were uncommon (1 time = 82.2%, 2 times = 15.4%, 3 times = 2.5%); therefore, we did not include the number of reactions in the analyses.

### 2.4 Statistical analyses

#### 2.4.1 Treatment effects on responsiveness, latency and duration

We conducted all statistical analyses using the function ‘glmer’ in the package ‘lme4’ (Bates et al., 2015) in R (4.3.2; R Development Core Team, 2021). We modelled all three focal variables as generalised linear mixed models (GLMMs). For responsiveness, we specified a GLMM with family ‘binomial’ (logit link), while for the latency and duration we used a GLMM with family ‘gamma’ (log link), which is suitable for continuous positive‐only data with positively skewed errors. This is because an a priori log‐transformation of the variables would not allow the interpretation of the estimates on an additive scale, which can substantially change any inference about interactive effects (Spake et al., 2023; see below).

We entered noise, light treatments and type of cue (acoustic vs. visual) to assess their main effects on tawny owls' focal behaviours. Furthermore, following the procedure described in Dominoni, Halfwerk, et al. (2020) and Dominoni, Smit, et al. (2020), we considered the combined effect of noise and light as a two‐way interaction. To disentangle the effect that each treatment has on behaviour in relation to specific prey cues, we also included a three‐way interaction between light treatment, noise treatment and type of cue for both responsiveness and hunting performance (i.e. latency and duration of response). This procedure allows for the exploration of the interactive effects (see below). We further considered the variable ‘year’ as a fixed factor. We included the same fixed effects in all three models. As random factors, we entered in all models individual ID to account for repeated measures of the same individual and the order the owls were tested during each night/trial, while we further entered the order of the prey cues only for the model exploring responsiveness. To test for parameter significance, we performed Wald Chi‐squared tests (ANOVA, type III).

To evaluate if there was habituation in focal behaviours due to treatment order, we pulled out the repeated individuals and ran simplified models with no interactions for each focal behaviour (same error distributions) where we additionally entered the type of trial (main vs. repetition) to compare treatment effects between main trials and repetitions. Finally, to further assess the consistency of owl behaviours, we ran three separate linear regressions to independently estimate the repeatability of responsiveness, latency and duration. The observations extracted from the repetitions were exclusively used to estimate the within‐individual consistency and habituation and were therefore not included in the main statistical analyses described above.

#### 2.4.2 Assessment of interactive effects of noise and light treatments

To identify type, magnitude and direction of the combined effects of noise and light treatments, we followed established studies that differentiate between additive, synergistic and antagonistic effects based on the observation of single and interactive effect sizes in relation to significant deviation from zero of the corresponding confidence intervals (Hale et al., 2017; Wilson et al., 2021). The interpretation of the effect sizes is scale specific and depends on whether the response variable is on an additive or multiplicative scale (Spake et al., 2023). To calculate a comparable effect size for all three focal variables, we ran separate models retaining only the two‐way interaction between noise and light treatments (Dominoni, Smit, et al., 2020; Smit et al., 2022) and used the functions ‘avg_comparisons’ and ‘comparisons’ in the package ‘marginaleffects’ (Arel‐Bundock, 2024), which compute and compare average marginal effects and conditional effects respectively for a variety of non‐linear link functions, allowing a hypothesis‐driven approach. Specifically, we tested whether the presence of light modifies the impact of noise on owl behaviour, calculating the confidence intervals of the effect sizes to account for the uncertainty of single and combined effects (Hale et al., 2017; Wilson et al., 2021). Single effects represent the magnitude of the deviation from the control expressed as a difference between slopes, whereas, for interactive effects, the ‘comparisons’ test returns the deviation from the additive expectation.

Accordingly, we classified the interactions as additive when the conditional effect did not significantly deviate from zero (i.e. no significant interaction), synergistic when the conditional effect was significant and in the same direction as the marginal effects, or antagonistic when the conditional effect was in the opposite direction to the stressor main effects. The presence of additivity would indicate that the two stressors operate independently of each other, a synergism would imply that the presence of light exacerbates the effect of noise, while an antagonism would occur when light buffers the effect of noise (Hale et al., 2017). We further calculated effect sizes separately for acoustic and visual cues to explore compounded effects of light and noise on each cue (Dominoni, Smit, et al., 2020; Smit et al., 2022).

## 3 RESULTS

Overall, we recorded 521 responses (217 (41.6%) to acoustic cues and 304 (58.2%) to visual cues) out of 1213 observations (42.9%), during which the owls never left the perch in pursuit of the mouse or the speakers. All owls responded at least once to the cues (Table S1). Our analysis showed that response in the repeat trials was predictive of a response during main trials, indicating that owl responsiveness was consistent over time (estimate ± SE = 0.55 ± 0.04, *t* = 13.02, *p* < 0.0001). Conversely, latency and duration were not statistically significant, suggesting they were not repeatable (estimate ± SE for latency: 0.15 ± 0.09, *t* = 1.63, *p* = 0.106, for duration: 0.02 ± 0.20, *t* = 0.10, *p* = 0.924). The models comparing the response in main versus repeated observations revealed that during the repetitions owls were less responsive (estimate ± SE for responsiveness = −0.33 ± 0.12, *z* = −2.72, *p* = 0.006), slower (estimate ± SE for latency = 0.33 ± 0.15, *t* = 2.15, *p* = 0.014) and less interested in the cues (estimate ± SE for duration = −0.25 ± 0.10, *t* = −2.44, *p* = 0.032) regardless of prey cue type. However, the patterns across treatments were comparable to those found for main trials (Figure S3a–c).

### 3.1 Responsiveness under noise and light treatments

**FIGURE 1:**
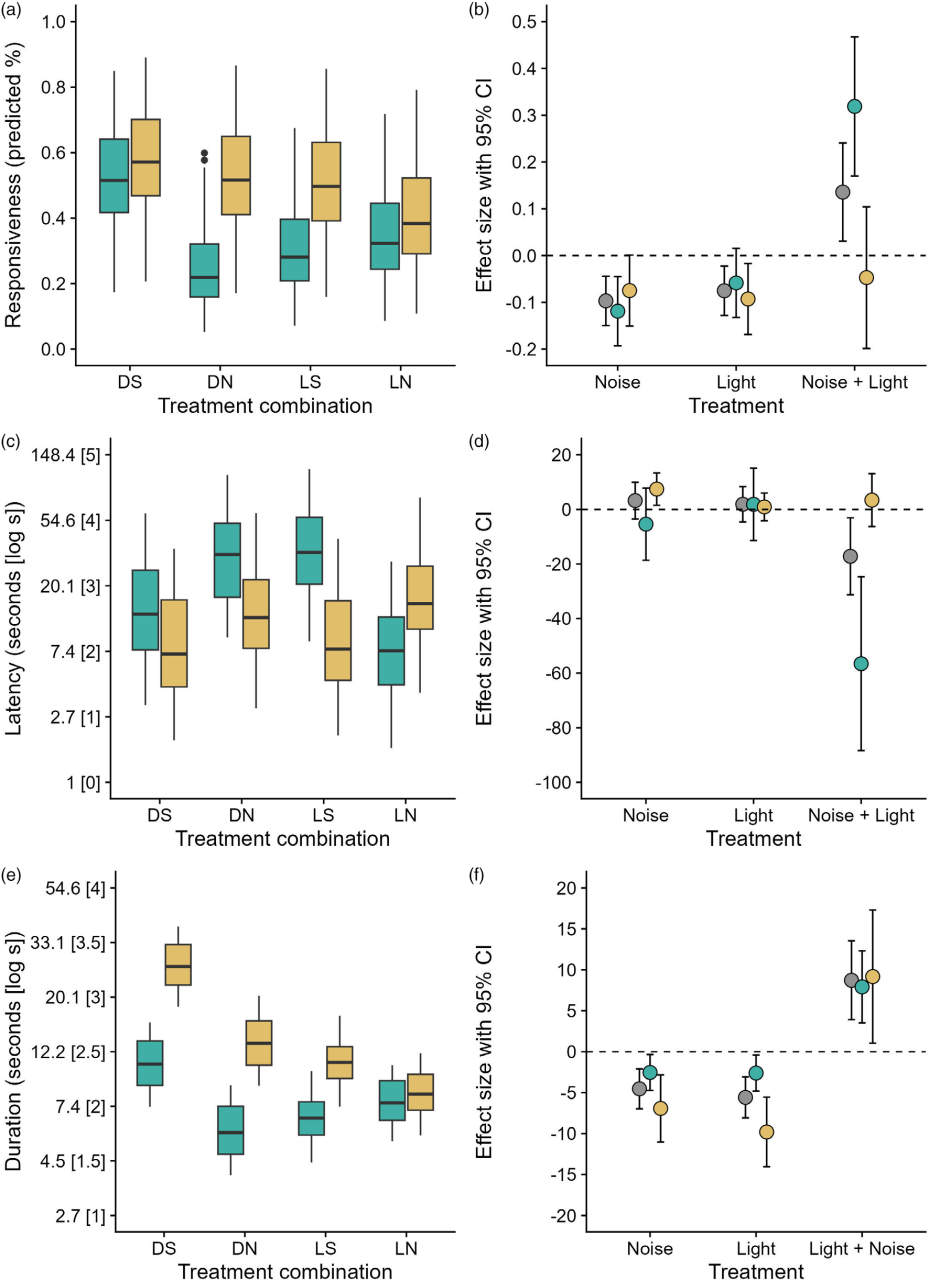
Single and combined effects of the exposure to noise and light on owl behaviour. On the left, boxplots depicting variation in (a) responsiveness, (c) latency and (e) duration of response in relation to the control (DS), noise (DN), light (LS) and noise + light (LN) treatments, for acoustic (green) and visual (yellow) cues. For latency and duration, values are log scaled (square brackets) with corresponding measures in seconds. One the right, bar plots showing marginal and conditional effects, with deviation from additive expectation (dashed lines), for the individual and combined effects of noise and light on responsiveness (b), latency (d) and duration (f). Effect sizes are shown for both acoustic and visual cues and, for comparison, also for both cues together (grey circles). Error bars indicate 95% CIs.

The model revealed a highly significant difference in responsiveness between control and manipulated conditions regardless of cue type, indicating that the exposure to noise (*χ* ^2^ = 25.39, df = 1, *p* < 0.0001) and light (*χ* ^2^ = 15.40, df = 1, *p* < 0.0001), either singularly or combined (*χ* ^2^ = 17.13, df = 1, *p* < 0.0001), negatively affects owl prey detection (Figure 1a). Cue type did not affect responsiveness (*χ* ^2^ = 0.31, df = 1, *p* = 0.580). Yet, when looking at the interaction between treatments and type of cue, we found that owls' ability to detect acoustic cues, not visual cues, was more strongly reduced by both noise and light (*χ* ^2^ = 11.68, df = 1, *p* = 0.001; Figure 1a). Responsiveness significantly differed between years, with owls in 2022 showing a higher response rate than owls in 2021 (*χ* ^2^ = 10.60, df = 1, *p* = 0.001; Figure S4a). For complete statistics, see Table S2a.

### 3.2 Marginal and conditional effects of noise and light on responsiveness

The average marginal effects confirmed that noise and light individually have a significant negative impact on owl responsiveness, reducing the probability to react by about 10% and 7% respectively (estimate noise = −0.097, 95% CI: −0.149, −0.044; estimate light = −0.075, 95% CI: −0.128, −0.022; Figure 1b). Conversely, the conditional effect showed that, when light is accounted for, the probability of reacting to stimuli under noisy conditions is 13% higher than expected under an additive expectation (estimate = 0.136, 95% CI: 0.031, 0.241; Figure 1b). Therefore, when no distinction between cues is made, the presence of light increases the likelihood of owl reaction.

When comparing marginal effects separately for acoustic cues, we found that light had a small non‐significant effect (estimate = −0.058, 95% CI: −0.132, 0.015), while noise had a twice as large negative effect (estimate = −0.119, 95% CI: −0.193, −0.045; Figure 1b). Yet, the conditional effect showed a 32% increase in the probability of reacting to the acoustic cues when light and noise were presented together (estimate = 0.319, 95% CI: 0.170, 0.467; Figure 1b), which suggests that the presence of light reduces the impact of noise on the response to acoustic stimuli.

For visual cues, we found that owls' likelihood to react was about 9% lower under light (estimate = −0.093, 95% CI: −0.169, −0.017) and 7% under noise, which, however, was only marginally significant (estimate = −0.075, 95% CI: −0.151, 0.001; Figure 1b). This might imply that the response to visual stimuli is less affected by noise. The conditional effect did not significantly deviate from an additive expectation (estimate = −0.047, 95% CI: −0.198, 0.104; Figure 1b). Noise and light therefore did not influence each other's effect on the response to visual stimuli. For complete statistics, see Table S3a–c.

### 3.3 Latency to respond under noise and light treatments

Among the owls which responded (Table S1), latency (*N* = 249 values across trials, visual cues =133 (53.4%), acoustic cues = 116 (46.6%)) was negatively affected by both noise (*χ* ^2^ = 7.68, df = 1, *p* = 0.006) and light (*χ* ^2^ = 10.88, df = 1, *p* = 0.001) and their interaction (*χ* ^2^ = 31.19, df = 1, *p* < 0.0001), indicating that owl detection was slower under manipulated conditions (Figure 1c). The cue type had no significant effect (*χ* ^2^ = 3.10, df = 1, *p* = 0.08). However, its interaction with treatments was highly significant (*χ* ^2^ = 20.89, df = 1, *p* < 0.0001), suggesting that owls were slower in detecting acoustic stimuli across treatments, whereas they responded consistently faster to visual cues (Figure 1c). Nonetheless, when exposed to combined noise and light, owls were faster in detecting acoustic cues (Figure 1c). Owls tested in 2022 were on average faster in detecting cues than owls in 2021 (year: *χ* ^2^ = 17.93, df = 1, *p* < 0.0001; Figure S4b). For complete statistics, see Table S2b.

### 3.4 Marginal and conditional effects of noise and light on latency

When considering both cues, we found that the effect sizes of both noise (estimate = 3.22, 95% CI: −0.350, 9.942) and light (estimate = 1.87, 95% CI: −4.610, 8.355) were rather small and with a wide uncertainty (Figure 1d). Conversely, the conditional effect was in the opposite direction and deviated significantly from an additive expectation (estimate = −17.19, 95% CI: −31.286, −3.101), showing that light in combination with noise quickened the response to cues by about 17 s.

When considering acoustic cues separately, the single effects were opposite but neither noise (estimate = −5.42, 95% CI: −18.636, 7.797) nor light (estimate = 1.85, 95% CI: −11.402, 15.101) significantly deviated from zero. Their combined effect, however, was much stronger than expected and decreased the delay in the response to acoustic stimuli of ca. 56 s (estimate = −56.55, 95% CI: −88.376, −24.716), indicating that owls performed better when exposed to noise and light together.

On the contrary, noise significantly delayed the onset of the response to visual cues (estimate = 7.44, 95% CI: 1.526, 13.348), whereas light did not have a significant effect (estimate = 0.90, 95% CI: −4.164, 5.972). The conditional effect was smaller than the single effect of noise, but did not deviate from zero (estimate = 3.39, 95% CI: −6.292, 13.075). Therefore, similarly to responsiveness, noise and light seemed to act independently on the timing of visual detection. For complete statistics, see Table S4a–c.

### 3.5 Duration of response to noise and light treatments

The duration of the response (*N* = 249; Table S1) was negatively affected by noise (*χ* ^2^ = 9.74, df = 1, *p* = 0.002), light (*χ* ^2^ = 8.33, df = 1, *p* = 0.004) and their combination (*χ* ^2^ = 8.81, df = 1, *p* = 0.003), indicating that owl interest in cues was lower under altered conditions (Figure 1e). Owls showed a longer duration of response to visual than to acoustic cues (*χ* ^2^ = 23.40, df = 1, *p* < 0.0001; Figure 1e; Figure S4c), but the three‐way interactive effect between cue type, noise and light treatments was not significant (*χ* ^2^ = 1.06, df = 1, *p* = 0.302). Year was not associated with variation in the duration of the response (*χ* ^2^ = 2.55, df = 1, *p* = 0.110). For complete statistics, see Table S2c.

### 3.6 Marginal and conditional effects of noise and light on duration of response

The marginal effects confirmed that noise (estimate = −4.528, 95% CI: −6.965, −2.092) and light (estimate = −5.577, 95% CI: −8.080, −3.075) significantly decreased the duration of the response. However, the conditional effect of noise and light combined was in the opposite direction and deviated significantly from zero, increasing the duration by about 8 s (estimate = 8.73, 95% CI: 3.909, 13.544; Figure 1f), indicating that the presence of light can enhance owl interest.

When considering cues separately, we found a similar negative pattern, although marginal effects were smaller for acoustic (estimate noise = −2.53, 95% CI: −4.725, −0.341; estimate light = −2.60, 95% CI: −4.808, −0.397) than for visual cues (estimate noise = −9.80, 95% CI: −14.053, −5.551; estimate light = −6.93, 95% CI: −11.031, −2.822; Figure 1f). The conditional effects were also comparable, with both acoustic (estimate = 7.92, 95% CI: 3.519, 12.313) and visual cues (estimate = 9.18, 95% CI: 1.038, 17.314; Figure 1f) deviating from the additive expectation. These findings suggest that mitigation effects on owl interest are independent of the cue type. For complete statistics, see Table S5a–c.

## 4 DISCUSSION

Noise and light pollution are expected to have considerable impacts on predator–prey interactions (Komine et al., 2020; Villalobos‐Jiménez et al., 2017). Our findings provide novel insights into the effects of noise and light pollution on the hunting behaviour of a nocturnal predator. Leveraging an experimental approach, we found that both noise and light impaired the owls' ability to detect the presence of potential prey and, when they detected the prey, light and noise delayed the response and affected owl interest in the potential prey. Slower response to detect cues may give prey more time to escape (Gomes et al., 2016) and thus affect hunting success. In agreement with our expectations, we also found that owls relied differently on acoustic and visual cues according to the treatment. While owls reacted equally to acoustic and visual cues in the absence of anthropogenic noise and light, acoustic detection was significantly more impaired than visual detection by both light and noise stressors. Against our predictions, the combined presence of noise and light did not favour visual cues. Instead, we observed an unexpected antagonistic interaction, with light mitigating the negative impact of noise on acoustic detection. Although habituation to the cues might be at play, as supported by a waning response across treatments confirmed in the repetitions, this finding does not appear to be influenced by the treatment order as owl prey detection varied across treatments in a similar fashion in the repetitions. This suggests that the effect exerted by noise and light together is genuine.

The masking effect of anthropogenic noise on the detection of biologically important acoustic cues is well documented and, together with distraction or disturbance, it impairs predator–prey dynamics and hunting performance (reviewed in Slabbekoorn et al., 2018). Our study confirms this general pattern and aligns with previous empirical research on owls and bats, where even moderate human‐made noise levels dramatically reduced the ability to locate prey acoustically, also far away from roads (Senzaki et al., 2016; Siemers & Schaub, 2011), and the chance of successful capture (Mason et al., 2016). However, fringe‐lipped bats (*Trachops cirrhosus*) have been found to actively compensate for such effects by relying on alternative cues like the inflating vocal sac of frogs, a strategy that bats did not show in the absence of masking noise (Gomes et al., 2016). Our findings show that similar coping strategies may arise also in owls as noise clearly compromised acoustic location, but did not affect visual detection, suggesting that masking and distraction effects might be contingent on the type of stimuli owls encounter and on owls' ability to shift to more usable cues for their foraging decision making. An increased use of visual cues might mitigate not only the negative effects of noise on prey detection but also on prey assessment and searching time (Taylor, 2009). This shift would thus avoid the effort of triangulating and localising prey sounds under noisy conditions with a higher risk of unsuccessful hunting (Senzaki et al., 2016). Nevertheless, Mason et al. (2016) found that noise affects all stages of hunting, including strikes and prey capture. Unfortunately, the lack of attacks on prey in this study prevents us from assessing whether a higher use of visual cues might compensate for noise‐induced striking failures as well.

Environmental light–dark cycle governs crucial behavioural and physiological processes and has shaped both predator preferences for specific prey cues and prey anti‐predator strategies (Gaston et al., 2013). By disrupting this cycle, light pollution could therefore mediate a preference for alternative cues previously underexploited (Davies & Smyth, 2018). For instance, toads and fast‐flying bats seem to benefit from the luring effect of ALAN on insects and increase their foraging activity around street lighting (Komine et al., 2020; Voigt et al., 2021). Here, we show that acoustic detection in tawny owls was severely affected by artificial light, whereas visual detection was less affected, which might underline an active effect of ALAN in favouring a shift towards sight‐oriented hunting strategies. Owls would therefore implement behaviours to directly rely on the presence of ALAN, such as higher foraging activity and attention to alternative prey cues (Rodríguez et al., 2021). Supporting this idea, we found higher response and interest for visual cues under light exposure. These behavioural adjustments in relation to night light changes might be linked to what nocturnal predators naturally experience owing to the lunar cycle (Gaston et al., 2013). In owls, moonlight drives an increased hunting effort during brighter nights to counter prey wariness (e.g. Clarke, 1983; San‐Jose et al., 2019). Evidently, while changes caused by moonlight are temporary albeit cyclic, ALAN produces a permanent change in the night‐time sensory landscape (i.e. ‘perpetual moonlight’; Longcore & Rich, 2004). Under this scenario, the expected owl response might be a complex trade‐off between the energetic investment in prey searching and the disruption in main prey behaviour due to ALAN (e.g. Rotics et al., 2011), possibly leading owls to target prey species easier to locate by sight (Rodríguez et al., 2021). This might widen owl trophic niche, but also have negative effects in terms of prolonged hunting effort, a possibility warranting further research.

Our study shows that owls consistently rely on visual cues under exposure to single sensory stressors. Nonetheless, the treatment combining noise and light did not provide conclusive support for our hypothesis that ALAN would favour sight. Indeed, we found an antagonistic effect of light on noise that seemed to ameliorate acoustic rather than visual detection. A possible explanation is that, when both stressors are present, light might affect how owls process noise stimuli (Dominoni, Smit, et al., 2020; Smit et al., 2022). For instance, light may provide a better view of the hunting grounds that, together with a potential increased hunting effort that light may elicit, might induce owls to heed any prey cue. In this regard, research on the barn owl (*Tyto alba*) brain showed that owls form a neural map of the auditory stimuli to precisely locate their prey that, however, requires visual inputs to register correctly the sounds and keep the map updated (Knudsen, 1982). This finding also affords an alternative interpretation of our results. Tawny owls' reliance on sight might be higher than previously thought for its importance in perceptual capabilities, which may explain why visual detection across treatments was mostly unaffected. Similarly to other nocturnal animals, owls have an exceptional scotopic vision (i.e. vision under dim‐light conditions; Martin, 1986), but can discriminate colours and UV spectra and move during the day as well, which suggests that they might switch between scotopic and photopic vision and maintain good visual performances under different light conditions (Orlowski et al., 2012). Exposure to sensory pollutants might thereby be neutral to visual system. Furthermore, owl perception of ALAN might vary according to the circumstances. Accordingly, while light could be attractive for hunting when other sources of disturbance are limited (Rodríguez et al., 2021), negative effects on owl distribution (Orlando & Chamberlain, 2023) might emerge for an association with high human presence rather than with a direct disrupting effect of light on hunting behaviour (Barrientos et al., 2023). Finally, other compensatory mechanisms may also arise over time, such as avoiding the overlapping with the highest noise levels (Gil et al., 2015) or individual variation in space use associated with particular morphological features such as the facial disc (Konishi, 1973)—the unique parabolic structure surrounding the owl's face in a majority of owl species—which enables acoustic detection independently of light.

Examining indirect and direct effects of noise and light in controlled environments is essential to fully understand the ecological impact of sensory pollutants on wildlife (Dominoni, Smit, et al., 2020; Gomes et al., 2016). To our knowledge, our study is the first to experimentally explore single and combined effects of noise and light pollution on hunting behaviour in avian nocturnal predators. However, experimental setups such as ours have limitations. In our experiment, we used naïve and inexperienced owls, which were raised in captivity and fed ad libitum and therefore possibly more used to visual than acoustic cues. Nevertheless, the responses were consistent over time and in line with studies on wild individuals (Mason et al., 2016; Senzaki et al., 2016). Additionally, our study did not allow us to assess tolerance thresholds for noise and light exposure. Considering the co‐occurrence of noise and light pollution, owl coping strategies might be context‐dependent and owls might aim to buffer the stressor that affects their hunting success the most (Senzaki, Barber, et al., 2020). Future studies should, for example, focus on the effects of ALAN and noise on space use, as nocturnal avian predators might show aversion for noise polluted areas (Fröhlich & Ciach, 2018) but still exploit artificially illuminated sites (Rodríguez et al., 2021).

Our research provides a first glimpse of coping strategies in owls hunting behaviour under noise‐ and light‐polluted conditions. Although we found only partial support for the idea that ALAN might promote visually oriented hunting strategies in combination with noise, we show that tawny owls were able to rely on the prey cue more suitable for the conditions they were experiencing, revealing that they might be able to mitigate the hampering effects of sensory stressors by switching to a different type of cue. This result highlights the complex interactive effects of ALAN and noise on (nocturnal) wildlife behaviour and predator–prey interplays and stresses the importance of more studies addressing this question to improve our understanding of the ecological impact of sensory pollutants, with positive implications for species conservation.

## AUTHOR CONTRIBUTIONS

Arianna Passarotto, Chiara Morosinotto and Patrik Karell conceived the study and designed the experiment. Arianna Passarotto performed the experiment and data extraction. Arianna Passarotto analysed the data with input from Chiara Morosinotto and Patrik Karell. Arianna Passarotto wrote the manuscript with help from all co‐authors.

## CONFLICT OF INTEREST STATEMENT

The authors have no conflict of interest to declare.

## STATEMENT OF INCLUSION

Our study brings together authors from different countries that were based at the institution where the study was carried out and was possible thanks to the help of scientists from the host institution and the collaboration with local ringers. All authors were engaged early on with the research and experimental design to ensure that the diverse sets of perspectives they represent were considered from the onset.

## Supporting information


**Table S1:** Table reporting the mean values with corresponding standard deviation (SD) for latency (left) and duration (right) for each treatment, type of prey cue and combination of treatments and prey cue (*N* = 249 total values).
**Table S2:** Results of the Binomial GLMM (a) and Gamma GLMMs (b, c) analyzing the effects of light and noise and their interaction on responsiveness (a), latency (b) and duration (c), respectively.
**Table S3:** Binomial GLMMs analyzing the two‐way interaction between noise and light on responsiveness considering all cues (a), only visual cues (b) and only acoustic cues (c).
**Table S4:** Gamma GLMMs, with log link function, analyzing the two‐way interaction between noise and light on latency considering all cues (a), only visual cues (b) and only acoustic cues (c).
**Table S5:** Gamma GLMMs, with log link function, analyzing the two‐way interaction between noise and light on duration of the response considering all cues (a), only visual cues (b) and only acoustic cues (c).
**Figure S1:** Satellite image showing the landscape context within a radius of approximately 4 km around the biological station (red pin).
**Figure S2:** Figure showing (A) part of the purpose‐built structure where the tawny owls were kept during the captive period. The red arrow indicates the position of the experimental aviary. The picture was taken in early winter, before the experiments started; (B) schematic drawing illustrating the experimental set‐up within the aviary: speakers to play acoustic cues (1), speaker to play traffic noise (2), dummy prey for visual cue (3), light spot to manipulate light conditions within the aviary (4) and infrared cameras to record owls' behaviour (5).
**Figure S3:** Boxplots comparing (a) responsiveness, (b) latency (log‐transformed) and (c) duration (log‐tranformed) between main trials (white boxes) and repetitions (grey boxes) across treatments, for acoustic (left panel—a) and visual (right panel—v) cues in repeated individuals (*N* =29).
**Figure S4:** Boxplots depicting the main effect of year on (a) the probability to react (i.e., responsiveness) and (b) latency (log‐trasformed).

## Data Availability

All data and codes to reproduce the analyses reported in this article are available from the Dryad Digital Repository: https://doi.org/10.5061/dryad.9zw3r22sq (Passarotto et al., 2025).
