# Supplementary material for: Experimental noise and light pollution alter prey detection in a nocturnal bird of prey
Source: J Anim Ecol. 2025 May 21;94(7):1398–409. doi: 10.1111/1365-2656.70062 (PMC12214444; doi:10.1111/1365-2656.70062)

Electronic supplementary material for

**Experimental noise and light pollution alter prey detection in a nocturnal bird of prey**

(Includes 5 tables and 4 figures)

**Table S1.** Table reporting the mean values with corresponding standard deviation (SD) for latency (left) and duration (right) for each treatment, type of prey cue and combination of treatments and prey cue (N = 249 total values). Number of responses for each specific case, as well as the number of owls which responded are also shown.

|  | **Mean latency (seconds)** | **±SD** | **Mean duration (seconds)** | **±SD** | **N responses** | **N individuals** |
| --- | --- | --- | --- | --- | --- | --- |
|  |  |  |  |  |  |  |
| Dark + Silence (DS) - control | 16.11 | 27.26 | 20.69 | 21.32 | 67 | 37 |
| Dark + Noise (DN) | 21.80 | 28.43 | 11.94 | 14.80 | 60 | 37 |
| Light + Silence (LS) | 24.40 | 33.48 | 9.03 | 6.86 | 62 | 36 |
| Light + Noise (LN) | 21.13 | 32.91 | 8.15 | 5.18 | 60 | 35 |
| Visual cue | 17.40 | 27.59 | 16.36 | 18.21 | 133 | 38 |
| Acoustic cue | 24.60 | 33.31 | 8.42 | 7.28 | 116 | 38 |
| Dark + Silence (DS) - visual cue | 12.32 | 23.96 | 29.54 | 26.26 | 33 | - |
| Dark + Noise (DN) - visual cue | 15.94 | 22.87 | 16.01 | 17.69 | 35 | - |
| Light + Silence (LS) - visual cue | 13.54 | 23.08 | 11.32 | 6.87 | 32 | - |
| Light + Noise (LN) - visual cue | 27.78 | 36.60 | 8.42 | 5.53 | 33 | - |
| Dark + Silence (DS) - acoustic cue | 19.79 | 30.02 | 12.11 | 9.30 | 34 | - |
| Dark + Noise (DN) - acoustic cue | 29.99 | 33.55 | 6.23 | 6.09 | 25 | - |
| Light + Silence (LS) - acoustic cue | 35.99 | 38.97 | 6.57 | 6.05 | 30 | - |
| Light + Noise (LN) - acoustic cue | 13.01 | 26.16 | 7.83 | 4.81 | 27 | - |
|  |  |  |  |  |  |  |

**Table S2.** Results of the Binomial GLMM (a) and Gamma GLMMs (b-c) analyzing the effects of light and noise and their interaction on responsiveness (a), latency (b) and duration (c), respectively. Models include noise and light treatment, type of cue and year as fxed terms. The contrast is given in brackets. ‘Individual ID’, ‘Order’, and ‘Order cue’ (for responsiveness) are entered as random factors. Bold font indicates statistically signifcant effects (α=0.05).

| a) | Dependent | Predictors | Estimate | SE | z | *P* |
| --- | --- | --- | --- | --- | --- | --- |
|  | Variable |  |  |  |  |  |
|  | Responsiveness | Intercept | -0.24 | 0.34 | -0.71 | 0.479 |
|  | (*n* = 1213) | **Treatment (Noise)** | **-1.33** | **0.26** | **-5.04** | **<0.0001** |
|  |  | **(Light)** | **-1.00** | **0.25** | **-3.92** | **<0.0001** |
|  |  | **(Light*Noise)** | **1.53** | **0.37** | **4.14** | **<0.0001** |
|  |  | **Treatment*Type of cue (Noise * Visual)** | **1.09** | **0.36** | **3.03** | **0.002** |
|  |  | (Light * Visual) | 0.68 | 0.35 | 1.93 | 0.054 |
|  |  | **(Light*Noise*Visual)** | **-1.74** | **0.51** | **-3.42** | **0.001** |
|  |  | Type of cue (Visual) | 0.25 | 0.45 | 0.55 | 0.580 |
|  |  | **Year (2022)** | **0.63** | **0.19** | **3.28** | **0.001** |
|  | Random factors | Individual ID: Variance 0.25, SD 0.50 |  |  |  |  |
|  |  | Order: Variance <0.0001 |  |  |  |  |
|  |  | Order cue: Variance 0.28, SD 0.53 |  |  |  |  |
| b) | Dependent | Predictors | Estimate | SE | t | *P* |
|  | Variable |  |  |  |  |  |
|  | Latency | **Intercept** | **3.14** | **0.26** | **11.94** | **<0.0001** |
|  | (*n* = 249) | **Treatment (Noise)** | **0.91** | **0.33** | **2.77** | **0.006** |
|  |  | **(Light)** | **1.01** | **0.31** | **3.30** | **0.001** |
|  |  | **(Light*Noise)** | **-2.68** | **0.48** | **-5.59** | **<0.0001** |
|  |  | Treatment*Type of cue (Noise * Visual) | -0.39 | 0.44 | -0.89 | 0.374 |
|  |  | **(Light * Visual)** | **-0.90** | **0.43** | **-2.10** | **0.003** |
|  |  | **(Light*Noise*Visual)** | **2.81** | **0.61** | **4.57** | **<0.0001** |
|  |  | Type of cue (Visual) | -0.54 | 0.31 | -1.76 | 0.078 |
|  |  | **Year (2022)** | **-1.06** | **0.25** | **-4.24** | **<0.0001** |
|  | Random factors | Individual ID: Variance 0.56, SD 0.75 |  |  |  |  |
|  |  | Order: Variance 0.003, SD 0.06 |  |  |  |  |
| c) | Dependent | Predictors | Estimate | SE | t | *P* |
|  | Variable |  |  |  |  |  |
|  | Duration | **Intercept** | **2.26** | **0.17** | **13.49** | **<0.0001** |
|  | (*n* = 249) | **Treatment (Noise)** | **-0.63** | **0.20** | **-3.12** | **0.002** |
|  |  | **(Light)** | **-0.55** | **0.19** | **-2.89** | **0.004** |
|  |  | **(Light*Noise)** | **0.84** | **0.28** | **2.97** | **0.003** |
|  |  | Treatment*Type of cue (Noise * Visual) | -0.37 | 0.27 | -1.39 | 0.166 |
|  |  | (Light * Visual) | -0.06 | 0.27 | -0.22 | 0.826 |
|  |  | (Light*Noise*Visual) | -0.40 | 0.38 | -1.03 | 0.302 |
|  |  | **Type of cue (Visual)** | **0.88** | **0.18** | **4.84** | **<0.0001** |
|  |  | Year (2022) | 0.24 | 0.15 | 1.60 | 0.110 |
|  | Random factors | Individual ID: Variance 0.08, SD 0.27 |  |  |  |  |
|  |  | Order: Variance 0.01, SD 0.09 |  |  |  |  |

**Table S3.** Binomial GLMMs analyzing the two-way interaction between noise and light on responsiveness considering all cues (a), only visual cues (b) and only acoustic cues (c). The contrast is given in brackets. ‘Individual ID’, ‘Order’, ‘Order cue’ are entered as random factors. Bold font indicates statistically signifcant effects (α=0.05).

|  |  | Predictors | Estimate | SE | z | χ^2^ | *P (χ^2^)* |
| --- | --- | --- | --- | --- | --- | --- | --- |
|  |  |  |  |  |  |  |  |
| a) | All cues | Intercept | -0.45 | 0.32 | -1.40 | 1.96 | 0.162 |
|  | (*n* = 1213) | **Treatment (Noise)** | **-0.76** | **0.18** | **-4.25** | **18.03** | **<0.0001** |
|  |  | **(Light)** | **-0.65** | **0.18** | **-3.69** | **13.60** | **<0.0001** |
|  |  | **(Light*Noise)** | **0.62** | **0.25** | **2.45** | **6.02** | **0.014** |
|  |  | Type of cue (Visual) | 0.69 | 0.39 | 1.75 | 3.05 | 0.081 |
|  |  | **Year (2022)** | **0.66** | **0.20** | **3.26** | **10.62** | **0.001** |
|  | Random factors | Individual ID: Variance 0.24, SD 0.49 |  |  |  |  |  |
|  |  | Order: Variance <0.0001 |  |  |  |  |  |
|  |  | Order cue: Variance 0.28, SD 0.53 |  |  |  |  |  |
| b) | Visual cue | Intercept | -0.30 | 0.21 | -1.44 | 2.08 | 0.149 |
|  | (*n* = 605) | Treatment (Noise) | -0.23 | 0.25 | -0.94 | 0.88 | 0.349 |
|  |  | (Light) | -0.31 | 0.25 | -1.26 | 1.58 | 0.208 |
|  |  | (Light*Noise) | -0.21 | 0.35 | -0.61 | 0.37 | 0.543 |
|  |  | **Year (2022)** | **1.27** | **0.21** | **5.88** | **34.60** | **<0.0001** |
|  | Random factors | Individual ID: Variance 0.14, SD 0.38 |  |  |  |  |  |
|  |  | Order: Variance <0.0001 |  |  |  |  |  |
| c) | Acoustic cue | **Intercept** | 0.12 | 0.23 | 0.51 | 0.26 | 0.609 |
|  | (*n* = 608) | **Treatment (Noise)** | **-1.27** | **0.26** | **-4.90** | **24.05** | **<0.0001** |
|  |  | **(Light)** | **-0.95** | **0.25** | **-3.82** | **14.62** | **<0.0001** |
|  |  | **(Light*Noise)** | **1.46** | **0.36** | **4.03** | **16.26** | **<0.0001** |
|  |  | Year (2022) | -0.07 | 0.26 | -0.27 | 0.08 | 0.784 |
|  | Random factors | Individual ID: Variance 0.34, SD 0.59 |  |  |  |  |  |
|  |  | Order: Variance <0.0001 |  |  |  |  |  |

**Table S4.** Gamma GLMMs, with log link function, analyzing the two-way interaction between noise and light on latency considering all cues (a), only visual cues (b) and only acoustic cues (c). The contrast is given in brackets. ‘Individual ID’ and ‘Order’ are entered as random factors. Bold font indicates statistically signifcant effects (α=0.05).

|  |  | Predictors | Estimate | SE | t | χ^2^ | *P (*χ^2^*)* |
| --- | --- | --- | --- | --- | --- | --- | --- |
|  |  |  |  |  |  |  |  |
| a) | All cues | **Intercept** | **3.11** | **0.23** | **13.39** | **179.34** | **<0.0001** |
|  | (*n* = 249) | **Treatment (Noise)** | **0.62** | **0.23** | **2.73** | **7.45** | **0.006** |
|  |  | **(Light)** | **0.57** | **0.22** | **2.53** | **6.40** | **0.011** |
|  |  | **(Light*Noise)** | **-0.89** | **0.34** | **-2.66** | **7.09** | **0.008** |
|  |  | **Type of cue (Visual)** | **-0.55** | **0.17** | **-3.22** | **10.38** | **0.001** |
|  |  | **Year (2022)** | **-0.92** | **0.25** | **-3.69** | **13.62** | **<0.0001** |
|  | Random factors | Individual ID: Variance 0.58, SD 0.76 |  |  |  |  |  |
|  |  | Order: Variance <0.0001 |  |  |  |  |  |
| b) | Visual cue | **Intercept** | **2.57** | **0.30** | **8.49** | **72.14** | **<0.0001** |
|  | (*n* = 133) | Treatment (Noise) | 0.42 | 0.24 | 1.74 | 3.03 | 0.082 |
|  |  | (Light) | -0.08 | 0.25 | -0.34 | 0.11 | 0.736 |
|  |  | (Light*Noise) | 0.23 | 0.33 | 0.68 | 0.46 | 0.496 |
|  |  | **Year (2022)** | **-1.21** | **0.33** | **-3.62** | **13.10** | **<0.0001** |
|  | Random factors | Individual ID: Variance 0.72, SD 0.85 |  |  |  |  |  |
|  |  | Order: Variance 0.11, SD 0.33 |  |  |  |  |  |
| c) | Acoustic cue | **Intercept** | **2.78** | **0.31** | **9.12** | **83.09** | **<0.0001** |
|  | (*n* =116) | **Treatment (Noise)** | **0.94** | **0.35** | **2.72** | **7.39** | **0.007** |
|  |  | **(Light)** | **1.11** | **0.33** | **3.38** | **11.45** | **0.001** |
|  |  | **(Light*Noise)** | **-2.70** | **0.51** | **-5.25** | **27.56** | **<0.0001** |
|  |  | **Year (2022)** | **-0.74** | **0.33** | **-2.27** | **5.17** | **0.023** |
|  | Random factors | Individual ID: Variance 0.67, SD 0.82 |  |  |  |  |  |
|  |  | Order: Variance <0.0001 |  |  |  |  |  |

**Table S5.** Gamma GLMMs, with log link function, analyzing the two-way interaction between noise and light on duration of the response considering all cues (a), only visual cues (b) and only acoustic cues (c). The contrast is given in brackets. ‘Individual ID’ and ‘Order’ are entered as random factors. Bold font indicates statistically signifcant effects (α=0.05).

|  |  | Predictors | Estimate | SE | t | χ^2^ | *P (*χ^2^*)* |
| --- | --- | --- | --- | --- | --- | --- | --- |
|  |  |  |  |  |  |  |  |
| a) | All cues | **Intercept** | **2.41** | **0.15** | **15.78** | **249.12** | **<0.0001** |
|  | (*n* = 249) | **Treatment (Noise)** | **-0.63** | **0.14** | **-4.55** | **20.73** | **<0.0001** |
|  |  | **(Light)** | **-0.74** | **0.14** | **-5.44** | **29.56** | **<0.0001** |
|  |  | **(Light*Noise)** | **0.62** | **0.20** | **3.18** | **10.12** | **0.001** |
|  |  | **Type of cue (Visual)** | **0.57** | **0.10** | **5.69** | **32.42** | **<0.0001** |
|  |  | Year (2022) | 0.24 | 0.15 | 1.58 | 2.51 | 0.113 |
|  | Random factors | Individual ID: Variance 0.08, SD 0.28 |  |  |  |  |  |
|  |  | Order: Variance 0.01, S.D. 0.10 |  |  |  |  |  |
| b) | Visual cue | **Intercept** | **2.87** | **0.18** | **15.63** | **244.43** | **<0.0001** |
|  | (*n* = 133) | **Treatment (Noise)** | **-0.59** | **0.18** | **-3.32** | **11.05** | **0.001** |
|  |  | **(Light)** | **-0.83** | **0.18** | **-4.57** | **20.93** | **<0.0001** |
|  |  | (Light*Noise) | 0.37 | 0.26 | 1.43 | 2.05 | 0.153 |
|  |  | **Year (2022)** | **0.55** | **0.18** | **2.99** | **8.94** | **0.003** |
|  | Random factors | Individual ID: Variance 0.09, SD 0.31 |  |  |  |  |  |
|  |  | Order: Variance <0.0001 |  |  |  |  |  |
| c) | Acoustic cue | **Intercept** | **2.50** | **0.19** | **12.93** | **167.08** | **<0.0001** |
|  | (*n* =116) | **Treatment (Noise)** | **-0.77** | **0.20** | **-3.97** | **15.74** | **<0.0001** |
|  |  | **(Light)** | **-0.72** | **0.19** | **-3.88** | **15.05** | **<0.0001** |
|  |  | **(Light*Noise)** | **1.00** | **0.27** | **3.65** | **13.34** | **<0.0001** |
|  |  | Year (2022) | -0.12 | 0.20 | -0.63 | 0.392 | 0.531 |
|  | Random factors | Individual ID: Variance 0.10, SD 0.33 |  |  |  |  |  |
|  |  | Order: Variance 0.01, S.D. 0.12 |  |  |  |  |  |

**Figure S1.** Satellite image showing the landscape context within a radius of approximately 4 km around the biological station (red pin). In the top left box, a zoomed image of the immediate surroundings of the field station (ca. 100 m radius), which is reached only by a country road without artificial illumination and is about 3 km from the closest village (Torna Hällestad) and ca. 4 km from the highway. The field station is mainly surrounded by woodlands. See “Study system” in the main text for a more complete description.

**
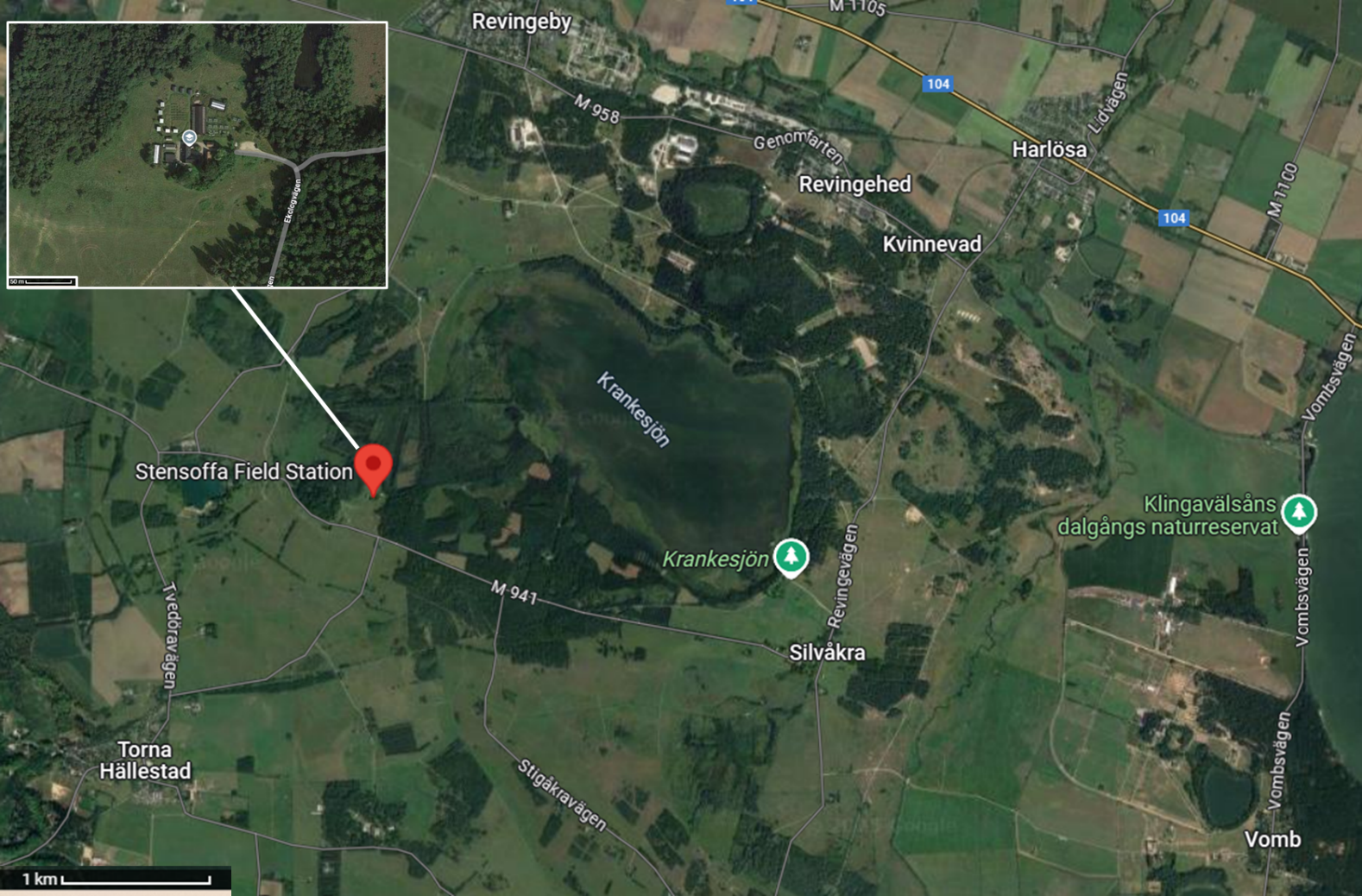
**

**Figure S2.** Figure showing A) part of the purpose-built structure where the tawny owls were kept during the captive period. The red arrow indicates the position of the experimental aviary. The picture was taken in early winter, before the experiments started; B) schematic drawing illustrating the experimental set-up within the aviary: speakers to play acoustic cues (1), speaker to play traffic noise (2), dummy prey for visual cue (3), light spot to manipulate light conditions within the aviary (4) and infrared cameras to record owls’ behaviour (5). For simplicity, only two perches are shown (see description in “Study system” in the main text).


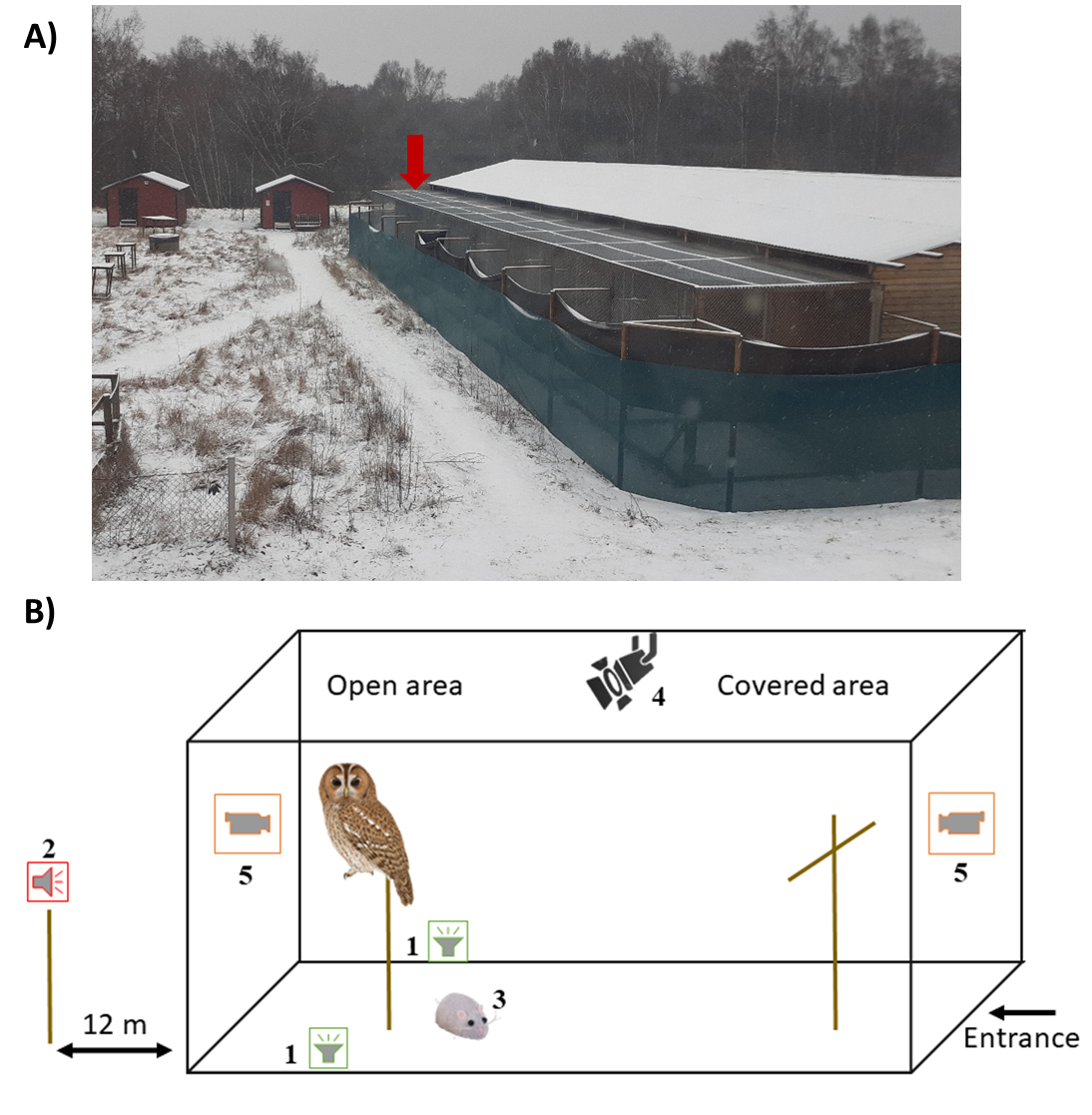


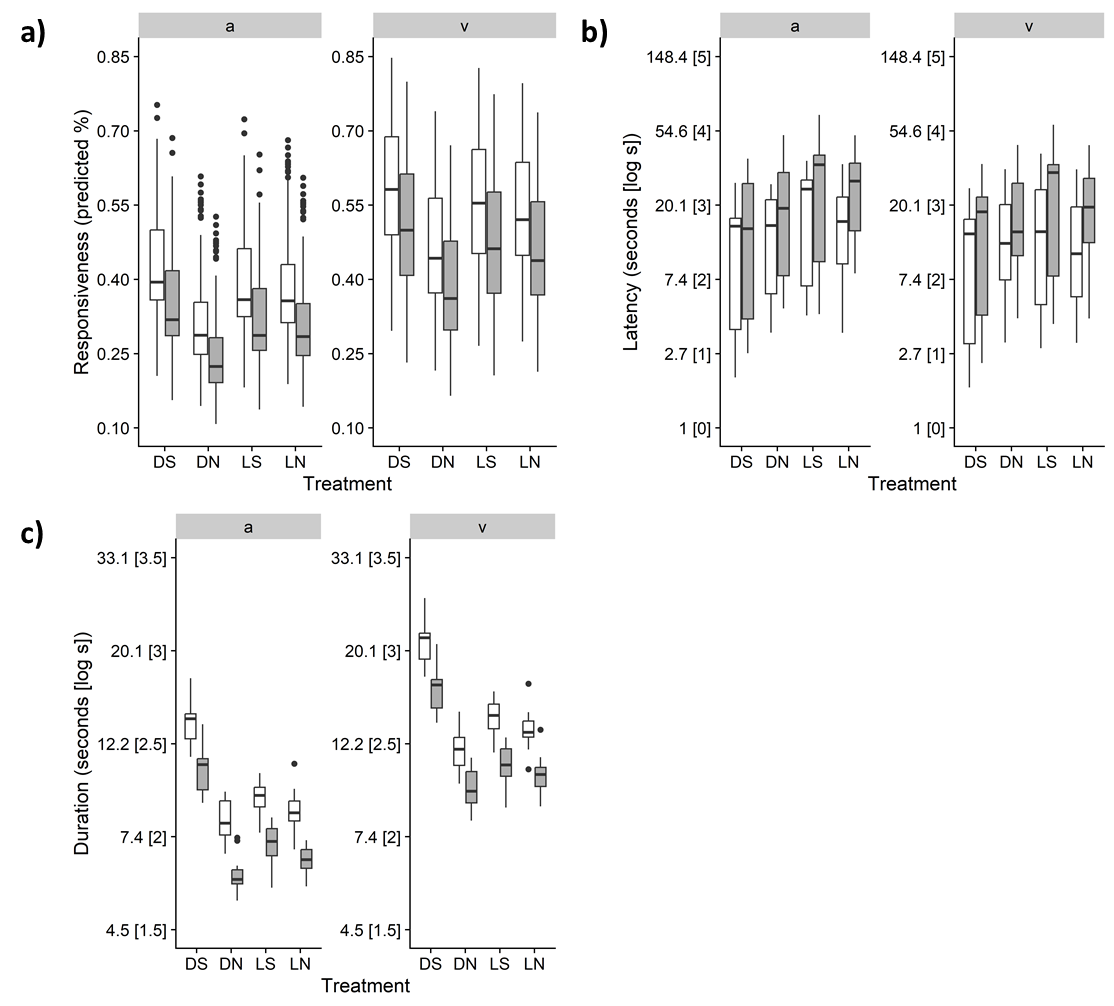
**Figure S3.** Boxplots comparing (a) responsiveness, (b) latency (log-transformed) and (c) duration (log-tranformed) between main trials (white boxes) and repetitions (grey boxes) across treatments, for acoustic (left panel - a) and visual (right panel - v) cues in repeated individuals (N =29).

**Figure S4.** Boxplots depicting the main effect of year on (a) the probability to react (i.e., responsiveness) and (b) latency (log-trasformed). In addition, (c) shows the main effect of type of cue on the duration of the response (log-trasformed).


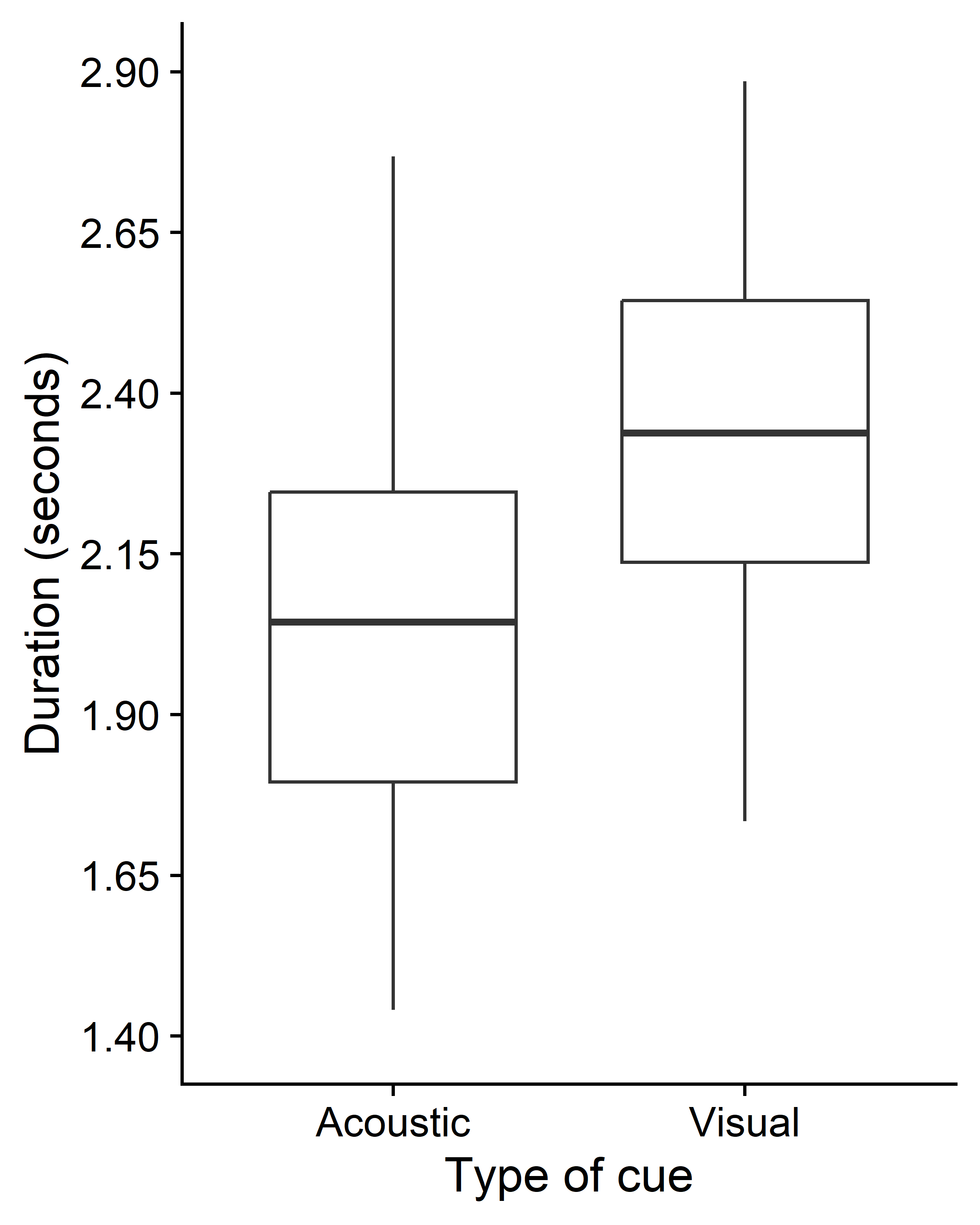

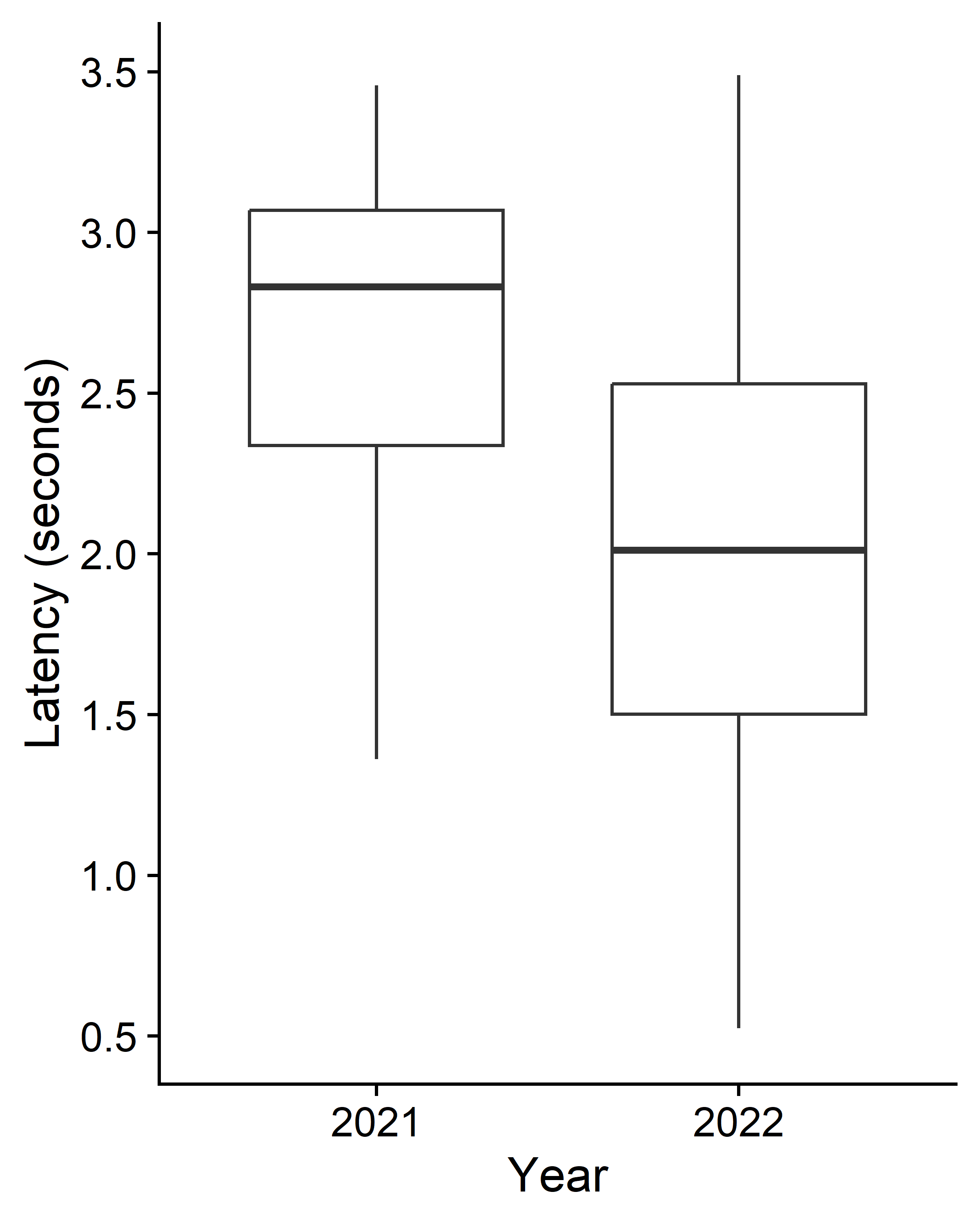


**c)**


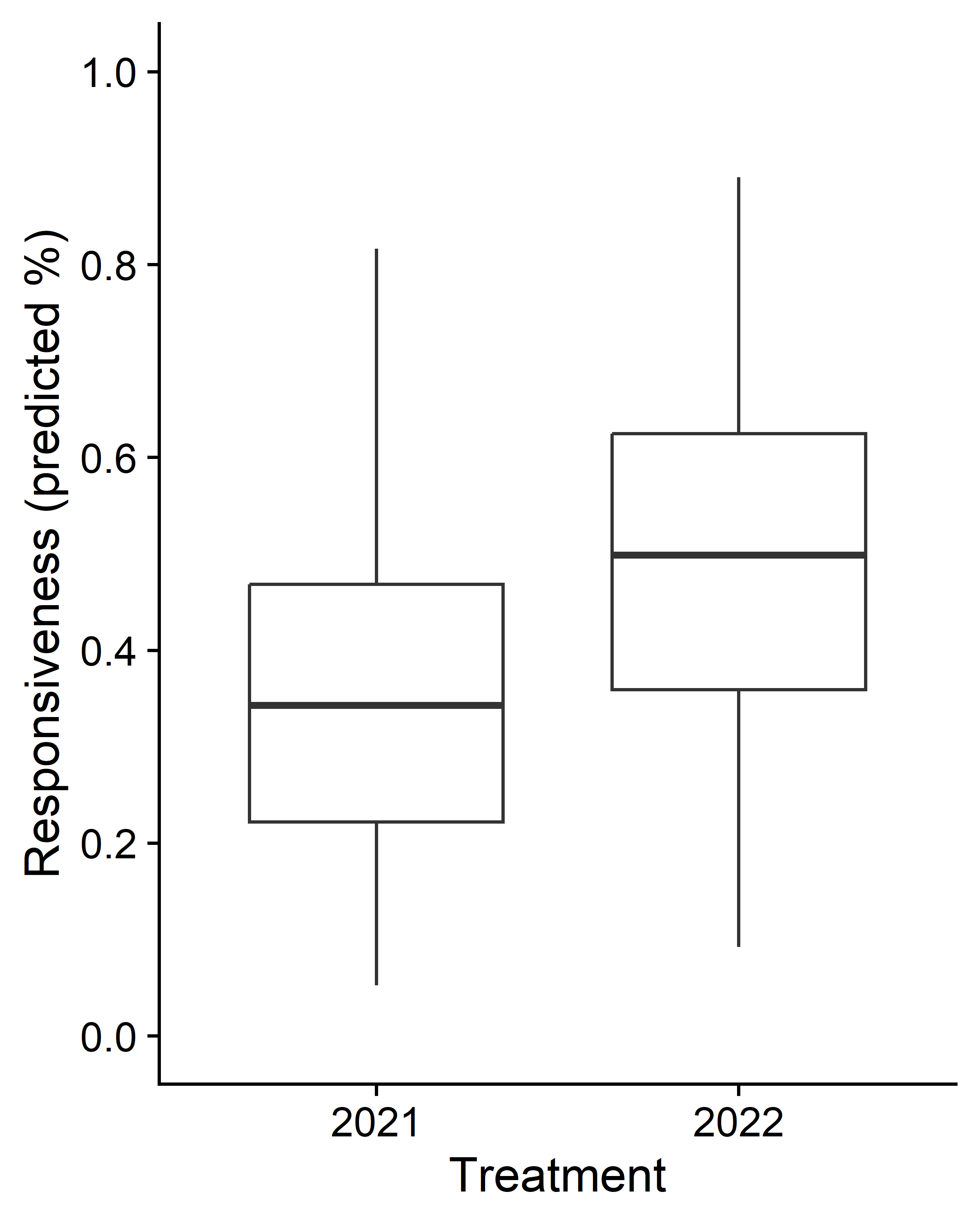


**a)**

**b)**


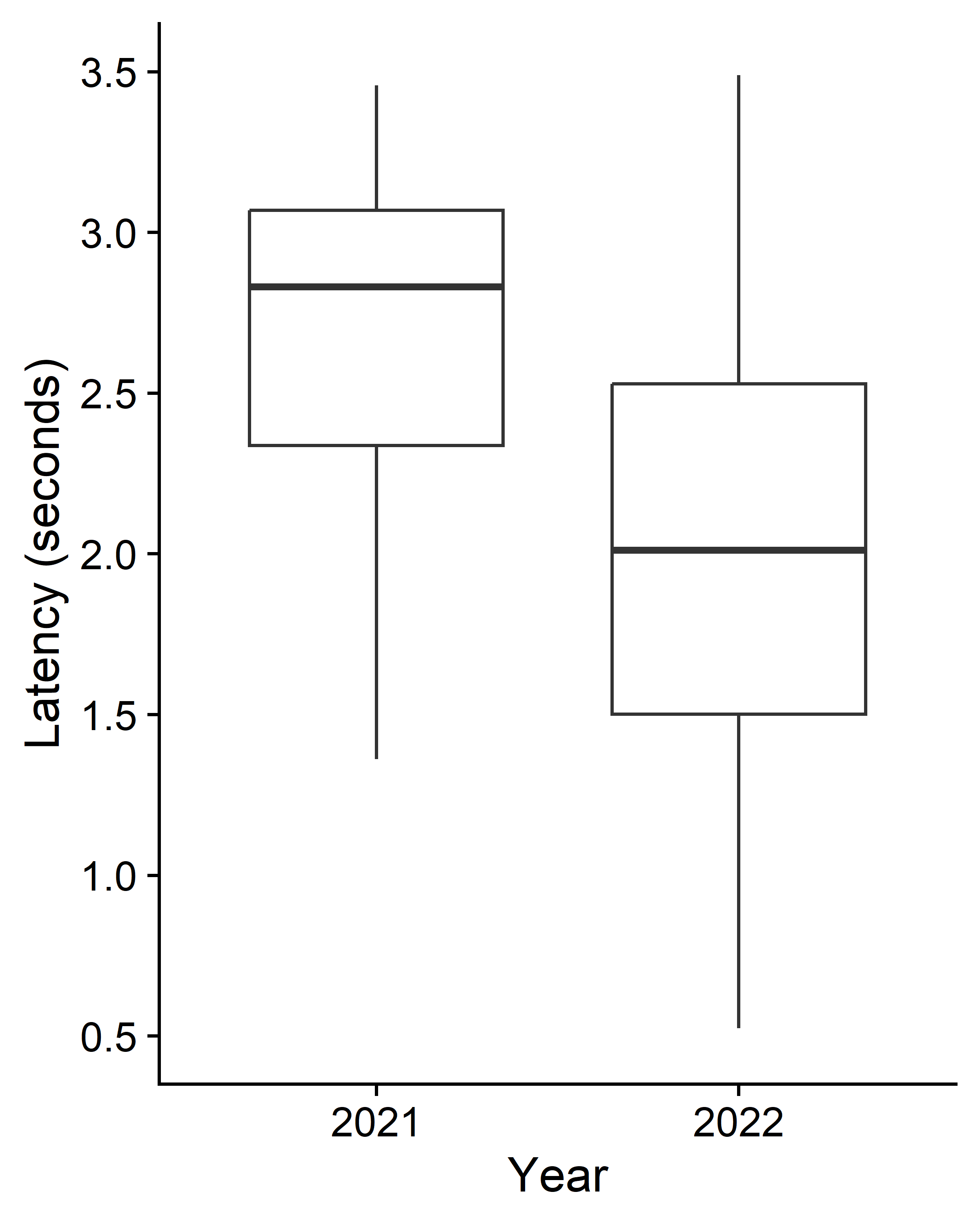

Supplement: Supplementary file 1 — Table S1: Table reporting the mean values with corresponding standard deviation (SD) for latency (left) and duration (right) for each treatment, type of prey cue and combination of treatments and prey cue (N = 249 total values). Table S2: Results of the Binomial GLMM (a) and Gamma GLMMs (b, c) analyzing the effects of light and noise and their interaction on responsiveness (a), latency (b) and duration (c), respectively. Table S3: Binomial GLMMs analyzing the two‐way interaction between noise and light on responsiveness considering all cues (a), only visual cues (b) and only acoustic cues (c). Table S4: Gamma GLMMs, with log link function, analyzing the two‐way interaction between noise and light on latency considering all cues (a), only visual cues (b) and only acoustic cues (c). Table S5: Gamma GLMMs, with log link function, analyzing the two‐way interaction between noise and light on duration of the response considering all cues (a), only visual cues (b) and only acoustic cues (c). Figure S1: Satellite image showing the landscape context within a radius of approximately 4 km around the biological station (red pin). Figure S2: Figure showing (A) part of the purpose‐built structure where the tawny owls were kept during the captive period. The red arrow indicates the position of the experimental aviary. The picture was taken in early winter, before the experiments started; (B) schematic drawing illustrating the experimental set‐up within the aviary: speakers to play acoustic cues (1), speaker to play traffic noise (2), dummy prey for visual cue (3), light spot to manipulate light conditions within the aviary (4) and infrared cameras to record owls' behaviour (5). Figure S3: Boxplots comparing (a) responsiveness, (b) latency (log‐transformed) and (c) duration (log‐tranformed) between main trials (white boxes) and repetitions (grey boxes) across treatments, for acoustic (left panel—a) and visual (right panel—v) cues in repeated individuals (N =29). Figure S4: Boxplots [file JANE-94-1398-s001.docx]
